# An Overview of *in vivo* Functions of Chondroitin Sulfate and Dermatan Sulfate Revealed by Their Deficient Mice

**DOI:** 10.3389/fcell.2021.764781

**Published:** 2021-11-24

**Authors:** Shuji Mizumoto, Shuhei Yamada

**Affiliations:** Department of Pathobiochemistry, Faculty of Pharmacy, Meijo University, Nagoya, Japan

**Keywords:** chondroitin sulfate, dermatan sulfate, epimerase, glycosyltransferase, knockout mouse, proteoglycan, sulfotransferase, transporter

## Abstract

Chondroitin sulfate (CS), dermatan sulfate (DS) and heparan sulfate (HS) are covalently attached to specific core proteins to form proteoglycans in their biosynthetic pathways. They are constructed through the stepwise addition of respective monosaccharides by various glycosyltransferases and maturated by epimerases as well as sulfotransferases. Structural diversities of CS/DS and HS are essential for their various biological activities including cell signaling, cell proliferation, tissue morphogenesis, and interactions with a variety of growth factors as well as cytokines. Studies using mice deficient in enzymes responsible for the biosynthesis of the CS/DS and HS chains of proteoglycans have demonstrated their essential functions. Chondroitin synthase 1-deficient mice are viable, but exhibit chondrodysplasia, progression of the bifurcation of digits, delayed endochondral ossification, and reduced bone density. DS-epimerase 1-deficient mice show thicker collagen fibrils in the dermis and hypodermis, and spina bifida. These observations suggest that CS/DS are essential for skeletal development as well as the assembly of collagen fibrils in the skin, and that their respective knockout mice can be utilized as models for human genetic disorders with mutations in chondroitin synthase 1 and DS-epimerase 1. This review provides a comprehensive overview of mice deficient in CS/DS biosyntheses.

## Introduction

**FIGURE 1:**
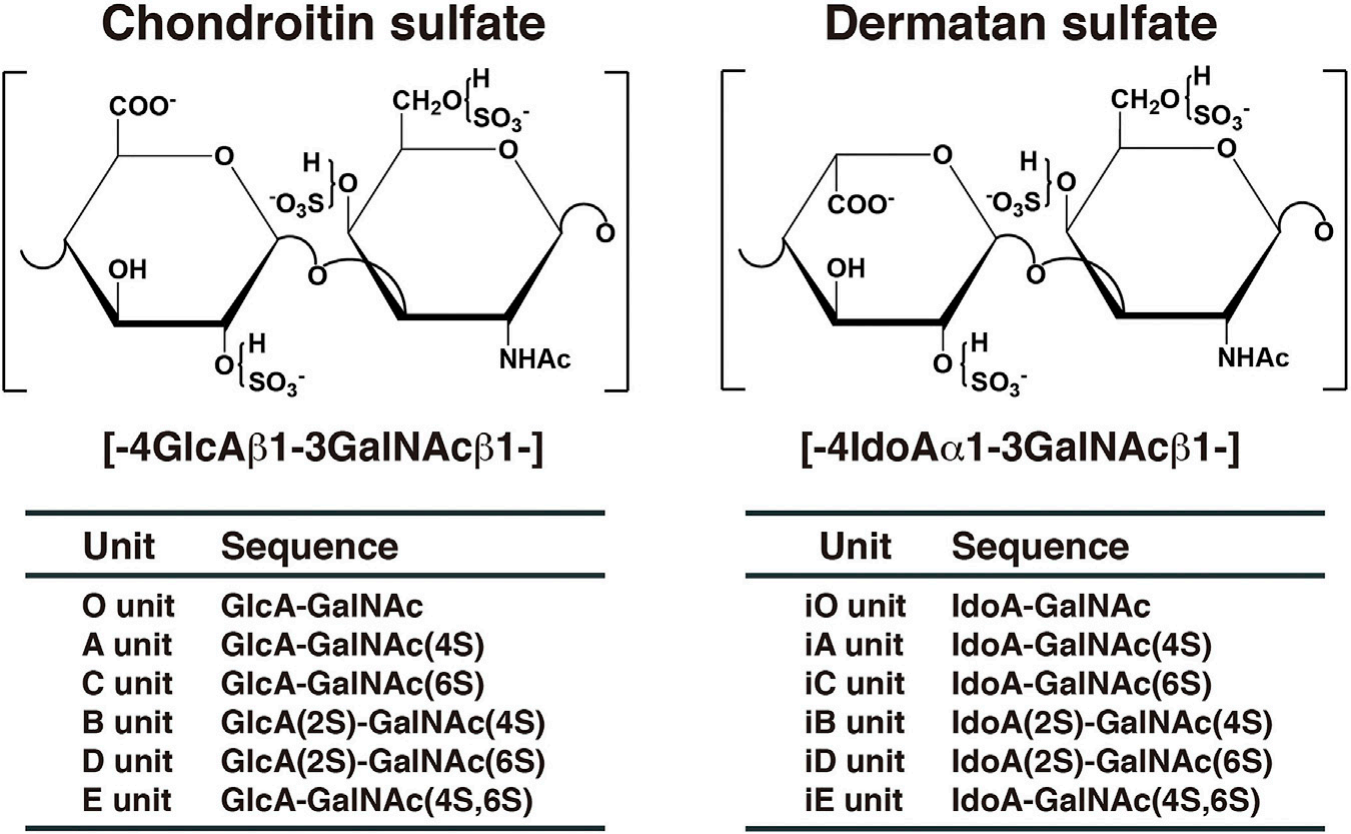
Typical repeating disaccharide units in CS and DS. CS consists of GlcA and GalNAc, whereas DS is a stereoisomer of CS including IdoA instead of GlcA. These sugar moieties are esterified by sulfate at various positions, as indicated in the figures.

Chondroitin sulfate (CS) and dermatan sulfate (DS) are covalently attached to core proteins to form proteoglycans (PGs). CS-PGs and DS-PGs are ubiquitously distributed in the extracellular matrix as well as on the cell surface (Rodén, 1980; Kjellén and Lindahl, 1991; Iozzo, 1998). Both glycosaminoglycans (GAGs) are linear polysaccharides. CS-PGs is abundantly distributed in cartilage (Rodén, 1980), whereas DS-PGs is predominantly distributed in skin, aorta, and blood vessel (Fransson et al., 1993). The backbone of CS is composed of repeating disaccharide units of D-glucuronic acid (GlcA) and *N*-acetyl-D-galactosamine (GalNAc) (Figure 1). DS is a stereoisomer of CS and consists of l-iduronic acid (IdoA) instead of GlcA and GalNAc (Figure 1). CS/DS chains are modified by sulfation at various hydroxy groups, which gives rise to structural diversity, thereby playing an important role in a variety of biological processes including interactions with various growth factors, cytokines, and morphogens, cell proliferation, tissue morphogenesis, and infections by viruses (Trowbridge and Gallo, 2002; Sugahara et al., 2003; Sugahara and Mikami, 2007; Malavaki et al., 2008; Yamada and Sugahara, 2008; Malmström et al., 2012; Thelin et al., 2013; Mizumoto et al., 2015; 2013; 2017; Mizumoto and Sugahara, 2013; Schaefer et al., 2017; Kosho et al., 2019). A variety of functions of CS/DS are thought to be dependent on sulfation modification (Sugahara and Mikami, 2007; Mizumoto et al., 2015). A, C, B, D, and E disaccharide units stand for the disaccharide (GlcA-GalNAc) units containing one or two sulfate groups in different combinations (Figure 1). If the GlcA residue has been epimerized to IdoA in each disaccharide unit, “i” is added to the codes, such as iA, iC, iB, iD, and iE (Figure 1). The A, iA, D, and E units are involved in infection of malaria, binding with heparin cofactor II, neurite outgrowth, and infection of herpes simplex virus, respectively (Maimone and Tollefsen 1991; Clement et al., 1998; Buffet et al., 1999; Bergefall et al., 2005). However, the functional domain in CS/DS does not appear to be composed of a single distinct saccharide sequence, but rather several heterogeneous sulfation patterns, the “wobble CS-DS motifs” (Purushothaman et al., 2012).

**FIGURE 2:**
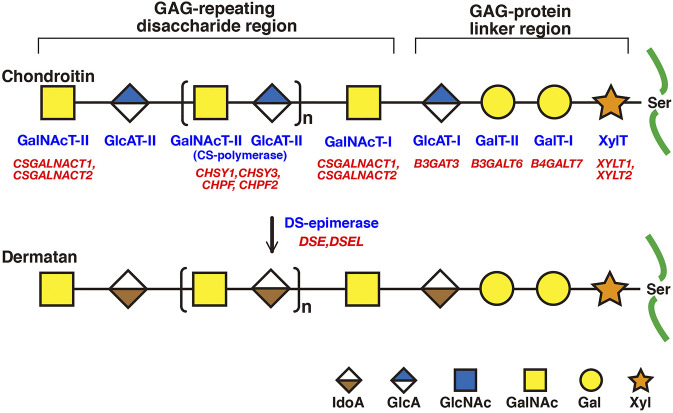
Biosynthetic assembly of CS and DS backbones by various glycosyltransferases. Schematic presentation of the biosynthesis of CS and DS backbones. All glycosyltransferases require a corresponding UDP-sugar, such as UDP-Xyl, -Gal, -GlcA, and -GalNAc, as a donor substrate. After specific core proteins have been translated, synthesis of the common GAG-protein linkage region, GlcAβ1-3Galβ1-3Galβ1-4Xylβ1-, is evoked by XylT, which transfers a Xyl residue from UDP-Xyl to the specific serine residue(s) at the GAG attachment sites. The linker region tetrasaccharide is subsequently constructed by GalT-I, GalT-II, and GlcAT-I. The first β1-4-linked GalNAc residue is then transferred to the GlcA residue in the linker region by GalNAcT-I, which initiates the assembly of the chondroitin backbone, thereby resulting in the formation of the repeating disaccharide region, [-4GlcAβ1-3GalNAcβ1-]_n_, by CS-polymerase. DS-epimerase converts GlcA into IdoA by epimerizing the C-5 carboxy group in the chondroitin precursor, thereby resulting in the formation of the repeating disaccharide region of dermatan precusor, [-4IdoAα1-3GalNAcβ1-]_n_. Each enzyme and its coding gene are described under the respective sugar symbols.

**FIGURE 3:**
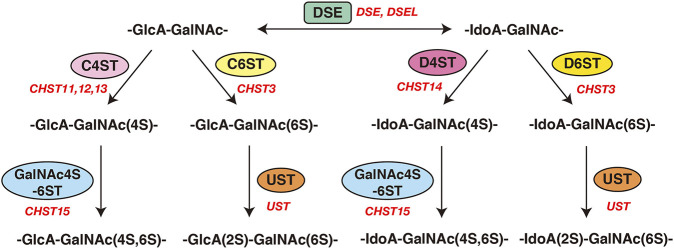
Modification of CS and DS by sulfotransferases and epimerases. Modification pathways of CS and DS. After formation of the CS/DS backbones, each sugar residue is modified by sulfation, catalyzed by sulfotransferases, as indicated in the figure. C4ST or C6ST transfers a sulfate group from PAPS to the C-4 or C-6 position of the GalNAc residues in the chondroitin chain, respectively. D4ST transfers a sulfate group from PAPS to the C-4 position of the GalNAc residues in dermatan. Further sulfation reactions are catalyzed by GalNAc4S-6ST or UST, which is required for formation of the disulfated disaccharide units indicated, respectively.

Various glycosyltransferases, epimerases, sulfotransferases, and related enzymes in the biosynthesis of CS and DS have been identified and characterized (Figures 2, 3) (Kusche-Gullberg and Kjellén, 2003; Mikami and Kitagawa, 2013; Mizumoto, 2018). Moreover, functional analyses of CS and DS using model organisms such as nematodes, fruit flies, zebrafish, and mice have revealed that both are indispensable for normal development (Sugahara and Schwartz, 1979; Bernhardt and Schachner, 2000; Hwang et al., 2003; Mizuguchi et al., 2003; Sugahara et al., 2003; Takemae et al., 2003; Olson et al., 2006; Maccarana et al., 2009; Mizumoto et al., 2009; Li et al., 2010; Tian et al., 2010; Watanabe et al., 2010; Wilson et al., 2012; Takemura et al., 2020). Genetic disorders related to mutations in biosynthetic enzymes for CS/DS-biosynthesis were described in another review article (Mizumoto and Yamada, 2021). This review focuses on recent advances in studies on mice deficient in CS and DS biosynthetic enzymes.

## Biosyntheses of CS and DS

### Biosyntheses of Donor Substrates for GAGs and Transporters of Uridine 5′-Diphosphate -Sugars, Sulfate Ions, and 3′-Phosphoadenosine 5′-Phosphosulfate

Most glycosyltransferases utilize uridine 5′-diphosphate (UDP)-sugars as the donor substrates, including: UDP-Glc, UDP-GlcNAc, UDP-GlcA, UDP-Gal, UDP-GalNAc, and UDP-Xyl, where Glc, GlcNAc, GlcA, Gal, GalNAc, and Xyl, represent D-glucose, *N*-acetyl-D-glucosamine, D-glucuronic acid, D-galactose, *N*-acetyl-D-galactosamine, and D-xylose, respectively. UDP-GlcA is formed by the action of UDP-Glc dehydrogenase on UDP-Glc in the cytosol (Table 1) (Spicer et al., 1998). UDP-Xyl is formed by the action of UDP-GlcA decarboxylase/UDP-xylose synthase in the endoplasmic reticulum and Golgi apparatus (Moriarity et al., 2002). These UDP-sugars mainly synthesized in the cytosol, except for UDP-Xyl, are incorporated into the endoplasmic reticulum and Golgi lumen through the corresponding nucleotide sugar transporters (Berninsone and Hirschberg, 2000; Orellana et al., 2016; Parker and Newstead, 2019).

**TABLE 1:** Transporters for UDP-sugars and sulfate, biosynthetic enzymes for PAPS and UDP-GlcA, and related proteins. Among the several transporters and biosynthetic enzymes involved in PAPS and UDP-sugars, GAG biosynthesis-related genes are listed here.

Transporters and enzymes	Coding genes	mRNA accession no	Phenotypes of KO or mutant mouse	Human genetic disorders	MIM number	Refs. For knockout mouse
UDP-glucose dehydrogenase	*Ugdh*	NM_009466	Defects in migration of mesoderm and endoderm, and disturbance of FGF signaling	Developmental and epileptic encephalopathy 84	603370	García-García and Anderson, (2003)
					618792	
PAPS synthase 2	*Papss2*	NM_001201470	A dome-shaped skull, reductions in limb size and axial skeletons, and disturbance of Indian hedgehog signaling	Brachyolmia 4 with mild epiphyseal and metaphyseal changes; Spondyloepimetaphyseal dysplasia Pakistani type (PAPSS2 type); Hyperandrogenism	612847	Orkin et al. (1976), Schwartz et al. (1978), Sugahara and Schwartz (1979), Sugahara and Schwartz (1982a), Sugahara and Schwartz (1982b), Pennypacker et al. (1981), Cortes et al. (2009)
		NM_001360403				
		NM_011864				
					603005	
Diastrophic dysplasia sulfate transporter (Solute carrier family 26 member A2)	*Slc26a2*	NM_007885	Growth retardation, joint contractures, and skeletal dysplasia including irregular size of chondrocytes, delay in the formation of the secondary osscification center, osteoporosis of long bone, severe thoracic kyphosis, bite overclosure, and hip dysplasia with pelvic deformity	Achondrogenesis type IB; Atelosteogenesis type II; De la Chapelle dysplasia; Diastrophic dysplasia; Diastrophic dysplasia, broad bone-platyspondylic variant; Epiphyseal dysplasia multiple 4	600972	Forlino et al. (2005)
					256050	
					222600	
					226900	
					606718	
UDP-GlcA/UDP-GalNAc dual transporter (Solute carrier family 35 member D1)	*Slc35d1*	NM_001356276	A lethal form of skeletal dysplasia including severe shortening of limbs, a decreased proliferating zone with round chondrocytes in the face, and scarce matrices	Schneckenbecken dysplasia	610804	Hiraoka et al. (2007)
		NM_177732			269250	
UDP 5′-diphosphatase	*Cant1*	NM_001025617	A moderate kyphosis, decrease in both length and width of tibiae, femurs, and ilium, delta phalanx, and a defect in endochondral ossification	Desbuquois dysplasia 1	617719	Paganini et al. (2019), Kodama et al. (2020)
		NM_001025618		Epiphyseal dysplasia multiple 7	251450	
		NM_001267591		Pseudodiastrophic dysplasia	613165	
		NM_001267592			264180	
		NM_029502				
3′-phosphoadenosine 5′-phosphate 3′-phosphatase	*Bpnt2/Impad1*	NM_177730	Either neonatal or embryonic lethality, reductions of limb length, shortening of the snout and lower limbs, and reduced sternal length	Chondrodysplasia with joint dislocations GRAPP type	614078	Frederick et al. (2008)
					614010	
Golgin, Rab6-interacting protein	*Gorab*	NM_001313738	Neonatal lethal. Abnormal collagen fibrils, thinned and porous cortical bone, and spontaneous fractures	Geroderma osteodysplasticum	607983	Chan et al. (2018)
		NM_178883			231070	

Cant1, calcium activated nucleotidase 1; Bpnt2, 3′(2′), 5′-bisphosphate nucleotidase 2; Impad1, inositol monophosphatase domain-containing protein 1; GRAPP, Golgi-resident phosphoadenosine phosphate phosphatase; MIM, mendelian inheritance in man.

Various GAG sulfotransferases catalyze the transfer of a sulfate group from 3′-phosphoadenosine 5′-phosphosulfate (PAPS), as a donor substrate, to respective acceptor substrates (Kusche-Gullberg and Kjellén, 2003). PAPS synthase (PAPSS) has two enzymatic domains, adenosine 5′-phosphosulfate kinase and ATP sulfurylase domains, in N- and C-terminals, respectively (Venkatachalam, 2003) (Table 1). PAPS is formed from inorganic sulfate, which is incorporated into the cytosol through the sulfate transporter at the plasma membrane and ATP (Hästbacka et al., 1994).

### Backbones of CS and DS

CS and DS polysaccharides are covalently attached to specific serine residues in core proteins through the common GAG-protein linker region tetrasaccharide GlcAβ1-3Galβ1-3Galβ1-4xylose(Xyl)β1-*O*- (Figure 2) (Lindahl and Rodén, 1972; Kjellén and Lindahl, 1991; Sugahara and Kitagawa 2000). The transfer of a Xyl residue from UDP-Xyl to specific serine residues in the newly synthesized core proteins of PGs in the endoplasmic reticulum and/or cis-Golgi compartments is initiated by β-xylosyltransferase (XylT) encoded by *XYLT1* or *XYLT2* (Figure 2; Table 2) (Götting et al., 2000; Pönighaus et al., 2007). It should be noted that human genes, which were described by all upper capital, were utilized in this section, because enzymatic activity of glycosyltransferases, epimerase, and sulfotransferases, which are responsible for biosynthesis of CS/DS, had been measured using recombinant human enzymes. β4-Galactosyltransferase-I (GalT-I) encoded by *B4GALT7*, then transfers a Gal residue from UDP-Gal to Xyl-*O*-serine in the core proteins (Almeida et al., 1999; Okajima et al., 1999). β3-Galactosyltransferase-II (GalT-II) encoded by *B3GALT6* transfers the second Gal residue from UDP-Gal to Gal-Xyl-*O*-serine (Bai et al., 2001). Thereafter, β3-glucuronyltransferase-I (GlcAT-I) encoded by *B3GAT3*, transfers a GlcA residue from UDP-GlcA to Gal-Gal-Xyl-*O*-serine (Figure 2; Table 2) (Kitagawa et al., 1998).

**TABLE 2:** Biosynthetic enzymes of the GAG-linkage region tetrasaccharide.

Enzymes	Coding genes	mRNA accession no	Phenotypes of KO or mutant mouse	Human genetic disorders	MIM numbers	Refs. For knockout mouse
Xylosytransferase	*Xylt1*	NM_175645	Reduced lengths of limb, humerus, femur, radius, ulna, tibia, and fibula, promotion of premature chondrocytes, and defect in endochondral ossification	Desbuquios dysplasia type 2; Short stature syndrome; Baratela-Scott syndrome	615777	Mis et al. (2014)
					608124	
					300881	
	*Xylt2*	NM_145828	Liver abnormalities including biliary tract hyperplasia, liver fibrosis, and biliary cysts, as well as renal abnormalities including dilated tubules, intestinal fibrosis, increase of renal weight, and hydronephrosis. Reductions in size and number of adipocytes, glucose intolerance, insulin resistance, and an increase in serum triglycerides	Spondyloocular syndrome	605822	Condac et al. (2007), Sivasami et al. (2019)
					608125	
β4Galactosyltransferase-I	*B4galt7*	NM_001311137	—	Ehlers-Danlos syndrome spondylodysplastic type 1; Ehlers-Danlos syndrome progeroid type 1; Ehlers-Danlos syndrome with a short stature and limb anomalies; Larsen of Reunion Island syndrome	130070	—
		NM_146045			604327	
β3Galactosyltransferase-II	*B3galt6*	NM_080445	—	Ehlers-Danlos syndrome spondylodysplastic type 2; Ehlers-Danlos syndrome progeroid type 2; Spondyloepimetaphyseal dysplasia with joint laxity type 1	615349	—
					615291	
					271640	
β3Glucuronyltransferase-I	*B3gat3*	NM_024256	An embryonic lethality before 8-cell stage	Multiple joint dislocations, a short stature, craniofacial dysmorphism with or without congenital heart defects	245600	Izumikawa et al. (2010), (2014)
				Larsen-like syndrome B3GAT3 type	606374	
				B3GAT3-related disorder with dislocation and congenital heart defects; B3GAT3-related disorder with cutis laxa and bone fragility; B3GAT3-related disorder with craniosynostosis and bone fragility; Pseudodiastrophic dysplasia	264180	
Glycosaminoglycan xylosylkinase	*Fam20b*	NM_145413	Underdifferentiation and overproliferation of chondrocytes, failure to initiate ossification on the popliteal side of the secondary ossification center, tongue elevation, micrognathia, microcephaly, suture widening, reduced mineralization in the calvaria, facial bones, and temporomandibular joint, death immediately after birth, marked intervertebral disc defects, and abnormal tooth development	Severe (lethal) neonatal short limb dysplasia with multiple dislocations	611063	Ma et al. (2016), Liu et al. (2018), Saiyin et al. (2019), Wu et al. (2020)
2-Phosphoxylose phosphatase 1	*Pxylp1*	NM_001289645	—	—	—	—
		NM_001289646				
		NM_001289647				
		NM_153420				

—, not reported; B4galt7, beta 1,4-galactosyltransferase 7; B3galt6, beta 1,3-galactosyltransferase 6; B3gat3, beta 1,3-glucuronyltransferase 3; Fam20b, Family with sequence similarity 20 member B.

Several modifications occur such as 2-*O*-phosphorylation and 2-*O*-dephosphorylation of Xyl and Xyl-2-*O*-phosphate residues by Xyl kinase and Xyl-2-*O*-phosphate phosphatase encoded by *FAM20B* and *PXYLP1*, respectively (Koike et al., 2009; 2014). Furthermore, sulfation at the C6 position of the first Gal and at C4 or C6 of the second Gal residues has been identified (Sugahara and Kitagawa, 2000). Chondroitin 6-*O*-sulfotransferase 1 (C6ST1) encoded by *CHST3* transfers a sulfate group from PAPS to Gal residues on the linker region tetrasaccharide GlcA-Gal-Gal-Xyl *in vitro* (Kitagawa et al., 2008). These modifications affect the glycosyltransferase reactions of GalT-I, GlcAT-I, CSGALNACT1, and may regulate the formation of CS/DS chains (Gulberti et al., 2005; Tone et al., 2008; Izumikawa et al., 2015).

Initiation of the repeating disaccharide region in the CS chain, [–4GlcAβ1–3GalNAcβ1–]_n_, is evoked by the transfer of the first GalNAc residue from UDP-GalNAc to the GlcA residue in the linker region tetrasaccharide, GlcA-Gal-Gal-Xyl-*O*-, by β4-*N*-acetylgalactosaminyltransferase-I (GalNAcT-I) encoded by *CSGALNACT1* or *CSGALNACT2* (Figure 2; Table 3) (Uyama et al., 2002; 2003). Chain elongation of CS occurs by the alternative addition of GlcA and GalNAc residues by CS-β3-glucuronyltransferase-II (CS-GlcAT-II) and GalNAcT-II, respectively (Figure 2; Table 3) (Mikami and Kitagawa, 2013). Chondroitin synthase (CHSY) encoded by *CHSY1* or *CHSY3* has a dual enzymatic activity of both CS-GlcAT-II and GalNAcT-II, which may be exerted in N- and C-terminal domains, respectively (Kitagawa et al., 2001b; Izumikawa et al., 2007). Chondroitin-polymerizing factor (CHPF) encoded by *CHPF* or *CHPF2* is able to construct the repeating disaccharide region of CS by forming an enzyme complex with CHSY (Kitagawa et al., 2003; Izumikawa et al., 2008). CHPF2 has both CS-GlcAT-II and GalNAcT-II activities; thereby, CHPF2 was designated as CHSY (Izumikawa et al., 2008). After or during construction of the non-sulfated disaccharide region of CS, the chondroitin backbone, it is modified by sulfation by the respective sulfotransferase including uronyl 2-*O*-sulfotransferase (UST) encoded by *UST* (Kobayashi et al., 1999), chondroitin 4-*O*-sulfotransferase (C4ST) encoded by *CHST11*, *CHST12*, or *CHST13* (Hiraoka et al., 2000; Yamauchi et al., 2000; Kang et al., 2002), C6ST encoded by *CHST3* (Fukuta et al., 1995; 1998), and GalNAc 4-*O*-sulfate 6-*O*-sulfotransferase (GalNAc4S-6ST) encoded by *CHST15* (Ohtake et al., 2001) (Figure 3; Table 3).

**TABLE 3:** Biosynthetic enzymes of CS and DS chains.

Enzymes (transferase activity)	Coding genes	mRNA accession no	Phenotypes of KO or mutant mouse	Human genetic disorders	MIM number	Refs. For knockout or transgenic mouse
Chondroitin sulfate synthase (GalNAcT-II, CS-GlcAT-II)	*Chsy1*	NM_001081163	Chondrodysplasia, progression of the bifurcation of digits, delayed endochondral ossification, reduced bone density, retinal stress, and decreased neutrophils in the bone marrow and spleen	Temtamy preaxial brachydactyly syndrome	605282	Wilson et al., 2012), Macke et al. (2020)
					608183	
	*Chsy3*	NM_001081328	A short body length and intervertebral disc degeneration	—	609963	Wei et al. (2020)
Chondroitin polymerizing factor	*Chpf*	NM_001001565	No obvious abnormalities, and slightly reduced length of femur and tibia	—	610405	Ogawa et al. (2012)
		NM_001001566				
	*Chpf2*	NM_133913	Anomalies of the bone and heart	—	608037	Tang et al. (2010)
Chondroitin sulfate *N*-acetylgalactosaminyltransferase (GalNAcT-I, GalNAcT-II)	*Csgalnact1*	NM_001252623	A short body length and small body weight caused by shorter limbs and axial skeleton, and a thinner growth plate in cartilage, impaired intramembranous ossification, malocclusion, abnormal eyes, skin hyperextension, severe scoliosis, joint laxity, and promotion of axonal regeneration after the spinal cord injury	Skeletal dysplasia, mild, with joint laxity and advanced bone age	616615	Watanabe et al. (2010), Sato et al. (2011), Takeuchi et al. (2013), Yoshioka et al. (2017), Hou et al. (2017), Ida-Yonemochi et al. (2018), Inada et al. (2021)
		NM_001364256				
		NM_172753				
	*Csgalnact2*	NM_172753	Normal development, fertility, growth rates, and skeletal formation	—	616616	Shimbo et al. (2017)
		NM_030165				
Dermatan sulfate epimerase	*Dse*	NM_172508	A smaller body weight, thicker collagen fibrils in the dermis and hypodermis, kinked tail, impairment of directional migration of aortic smooth muscle cells, defects in fetal abdominal wall, exencephaly, and spina bifida	Ehlers-Danlos syndrome musculocontractural type 2	615539	Maccarana et al. (2009), Gustafsson et al. (2014), Bartolini et al. (2013), Stachtea et al. (2015)
					605942	
	*Dsel*	NM_001081316	Normal extracellular matrix features	Bipolar disorder; Depressive disorder; Diaphragmatic hernia; Microphthalmia	611125	Bartolini et al. (2012), Stachtea et al. (2015)
Chondroitin 6-*O*-sulfotransferase	*Chst3*	NM_016803	Decreased number of naive T-lymphocytes, hyperthickened *epidermis*, enhanced proliferation and altered differentiation of basal keratinocytes, few regenerating axons, and more axonal retraction after axotomy of nigrostriatal axons	Spondyloepiphyseal dysplasia with congenital joint dislocations; Spondyloepiphyseal dysplasia Omani type; Chondrodysplasia with multiple dislocations Megarbane type; Humerospinal dysostosis; Larsen syndrome autosomal recessive type; Desbuquois syndrome	143095	Uchimura et al. (2002), Lin et al. (2011), Properzi et al. (2005), Miyata et al. (2012), Kitazawa et al. (2021)
					603799	
Chondroitin 4-*O*-sulfotransferase	*Chst11*	NM_021439	Severe dwarfism, multiple skeletal abnormalities including a small rib cage, a kinked vertebral column, severely shortened limbs, and a dome-shaped skull, reduction in Alcian blue staining in cartilage, and died within 6 h of birth with severe respiratory distress	Osteochondrodysplasia, brachydactyly, and overlapping malformed digits	610128	Klüppel et al., 2005, Bian et al. (2011)
					618167	
Dermatan 4-*O*-sulfotransferase	*Chst14*	NM_028117	A smaller body mass, reduced fertility, kinked tail, increased skin fragility, disorganized collagen fibers, thoracic kyphosis, myopathy-related phenotypes including variation in fiber size and spread of the muscle interstitium, alterations in the vascular structure of the placenta, an abnormal structure of the basement membrane of capillaries in the placental villus, an increase of proliferation of Schwann cells, better recovery after femoral nerve injury, and a small number and large diameter of neurospheres	Ehlers-Danlos syndrome musculocontractural type 1; Ehlers-Danlos syndrome, type VIB; Adducted thumb-clubfoot syndrome	601776	Bian et al. (2011), Akyüz et al. (2013), Yoshizawa et al. (2018), Hirose et al. (2021), Nitahara-Kasahara et al. (2021a)
					608429	
*N*-Acetylgalactosamine-4-sulfate-6-*O*-sulfotransferase	*Chst15*	NM_001360768	Weak staining of bone marrow-derived mast cells with May Grünwald-Giemsa, increase in empty granules in bone marrow-derived mast cells, lower activities of carboxypeptidase A and tryptase from bone marrow-derived mast cells, low bone mass, impairment of osteoblast differentiation, and enhanced liver fibrosis induced by CCl_4_	—	608277	Ohtake-Niimi et al. (2010), Koike et al., (2015), Habuchi et al. (2016), Nadanaka et al. (2020)
		NM_029935				
Uronyl 2-*O*-sulfotransferase	*Ust*	NM_177387	—	Multiple congenital anomalies of the heart and central nervous system	610752	—

—, not reported; CHST, carbohydrate sulfotransferase.

**TABLE 4:** Outstanding questions and perspectives for functions of glycosyltransferases, sulfotransferases, and epimerase involving CS/DS-biosynthesis.

Questions	Related enzymes	Related references
How XYLTs recognize serine residues on core proteins?	XYLT1, XYLT2	Götting et al. (2000), Pönighaus et al. (2007)
What sorting mechanism of CS/DS and HS?	CSGALNACT1, CSGALNACT2, EXTL2, EXTL3	Izumikawa and Kitagawa (2015), Izumikawa et al. (2015), Koike et al. (2009), Koike et al. (2014), Sugahara and Kitagawa, (2000)
Which GalTs compensate GalT-I and GalT-II-deficiencies?	B4GALTs, B3GALTs	Almeida et al. (1999), Okajima et al. (1999), Bai et al. (2001), Mizumoto and Yamada (2021)
How three dimensional structures of glycosyltransferases and sulfotransferases?	CHSY1, CHPF, DSE, CHST14	Kitagawa et al. (2001a), Kitagawa et al. (2003), Maccarana et al. (2006), Evers et al. (2001), Mikami et al. (2003)
What is the differential roles of the respective isoforms?	XYLTs, CHSYs, CHPFs, CSGALNACTs, C4STs, DSEs	Götting et al. (2000), Kitagawa et al. (2001b), Kitagawa et al., 2003, Uyama et al. (2002), Hiraoka et al. (2000), Maccarana et al. (2006)
What is the roles of 2-O-sulfation in CS/DS?	UST	Kobayashi et al. (1999)
What is the roles of CS/DS in tumor metastasis and development?	All CS/DS-biosynthetic enzymes	ten Dam et al. (2007), Bi et al., (2008), Li et al. (2008), Sugahara et al. (2008), Mizumoto et al. (2012)
Which golgin(s) regurate GAG biosynthesis?	All CS/DS-biosynthetic enzymes	Chan et al. (2018), Ferreira et al. (2018)
Regulation of gene expression and related transcriptional factors	All genes encoding CS/DS-biosynthetic enzymes	Kitagawa et al. (2001a)

Formation of the repeating disaccharide region, [–4IdoAβ1–3GalNAcβ1–]_n_, of DS chains occurs by epimerization of the C5 position of GlcA residues in a chondroitin precursor backbone, which is catalyzed by DS-epimerase encoded by *DSE* or *DSEL* (Figure 2) (Maccarana et al., 2006; Pacheco et al., 2009). The dermatan chains are modified by sulfation catalyzed by UST and dermatan 4-*O*-sulfotransferase (D4ST) encoded by *UST* and *CHST14*, which transfer the sulfate from PAPS to the C2 position of IdoA and C4 position of GalNAc residues, respectively (Kobayashi et al., 1999; Evers et al., 2001; Mikami et al., 2003) (Figure 3; Table 3).

### Catabolism of Donor Substrates for CS/DS Biosynthesis

After glycosyltransferase reaction, the reaction product, UDP, derived from UDP-sugar is hydrolyzed into uridine 5′-monophosphate (UMP) by nucleoside 5′-diphosphatase, which is encoded by *calcium-activated nucleotidase 1* (*CANT1*), in the endoplasmic reticulum and Golgi apparatus (Table 1) (Failer et al., 2002; Smith et al., 2002). UMP is exported to the cytosol by nucleotide sugar transporters, which are antiporters for UDP-sugars and UMP, from the Golgi apparatus and/or endoplasmic reticulum (Parker and Newstead, 2019).

After the sulfotransferase reaction, the reaction product, adenosine-3′, 5′-bisphosphate (PAP), derived from PAPS is hydrolyzed into adenosine 5′-phosphate (5′-AMP) by the Golgi-resident PAP 3′-phosphatase, which is encoded by *3′(2′), 5′-bisphosphate nucleotidase 2* (*BPNT2*)*/inositol monophosphatase domain containing 1* (*IMPAD1*) (Table 1) (Frederick et al., 2008). The 5′-AMP may be exported to the cytosol by unidentified transporters from the Golgi apparatus and/or endoplasmic reticulum.

## Knockout and Mutant Mice of Biosynthetic Enzymes of CS/DS and Its Donor Substrates as Well as Nucleotide Sugar Transporters

### Ugdh

UDP-Glc dehydrogenase (UGDH) is an oxidoreductase that converts UDP-Glc to UDP-GlcA in the cytosol (Spicer et al., 1998). The mutant mice *lazy mesoderm* have a mutation in *Ugdh*, which was introduced by ethyl-nitrosourea, and show a phenotype of embryogenesis arrest during gastrulation with defects in migration of the mesoderm and endoderm (García-García and Anderson, 2003). Furthermore, no CS or heparan sulfate (HS) were detected in the mutant using respective antibodies against them (García-García and Anderson, 2003). HS is also linear polysaccharide of GAG family, and composed of repeating disaccharide unit, [-4GlcAβ1–4GlcNAcα1-]_n_, which is covalently attached to the specific core proteins, forming PGs (Supplemental Figure S1) (Kjellén and Lindahl, 1991). HS and HS-PGs play essential roles in signal transduction, tissue morphogenesis, early development, and tumor progression (Bishop et al., 2007). The disturbance of FGF signaling has been demonstrated in the *Ugdh* mutant, resulting in a similar phenotype to those of *Fgf8* and *Fgfr1* mutants (Yamaguchi et al., 1994; Sun et al., 1999). The interaction of not only HS but also CS with FGFs and their receptors has been shown to be required for signal transduction (Esko and Selleck, 2002; Bishop et al., 2007; Mizumoto et al., 2015). Thus, the phenotype of the *Ugdh* mutant might be caused by defects in HS and/or CS.

### Papss2

PAPS synthase (PAPSS) is a dual enzyme with both adenosine 5′-phosphosulfate kinase and ATP sulfurylase activities, catalyzed by its N- and C-terminal domains, respectively (Fuda et al., 2002; Venkatachalam, 2003). The *Papss2* mutant, brachymorphic mouse, which is generated by *N*-ethyl-*N*-nitrosourea, and has the substitution Gly79Arg, shows a normal life span, a dome-shaped skull, and reductions in limb as well as axial skeletons, thereby leading to brachymorphism (Schwartz et al., 1978; Sugahara and Schwartz, 1979, 1982a, 1982b; Pennypacker et al., 1981). Moreover, the mutant mice produce lower sulfated CS but not HS in the growth plate cartilage, and show disturbed Indian hedgehog signaling due to abnormal distribution in the extracellular matrix, which results in a reduction in chondrocyte proliferation (Orkin et al., 1976; Cortes et al., 2009). These findings suggest that the sulfation in CS side chains of PG(s), such as aggrecan, modulates Indian hedgehog signaling.

### Slc26a2

The sulfate transporter is encoded by *SLC26A2*, which incorporates a sulfate anion into the cytosol at the plasma membrane (Hästbacka et al., 1994; Satoh et al., 1998; Seidler and Nikolovska, 2019). The incorporated sulfate is activated to adenosine-phosphosulfate and then to PAPS by PAPS synthase (Venkatachalam, 2003). An *Slc26a2* knock-in mouse with an Ala386Val substitution in the eighth transmembrane domain of *Slc26a2*, whose mutation was detected in a patient with diastrophic dysplasia characterized by a short stature, cleft plate, and deformity of the external ear and thumb (Rossi and Superti-Furga, 2001), was characterized by growth retardation, joint contracture, and skeletal dysplasia including an irregular size of chondrocytes, delay in the formation of the secondary osscification center and osteoporosis of long bones, severe thoracic kyphosis, bite overclosure, and hip dysplasia with pelvic deformity (Forlino et al., 2005). Furthermore, the proportion of a non-sulfated disaccharide unit, GlcA-GalNAc, was higher than that of the wild-type in cartilage and bone, but not skin (Forlino et al., 2005). These findings suggest that abnormalities of proliferation and differentiation of chondrocytes contribute to reduced bone growth, and lead to similar phenotypes to probands of human diastrophic dysplasia. Thus, this mutant mouse is a useful model to explore the pathogenic and therapeutic approaches for human diastrophic dysplasia.

### Slc35d1

UDP-GlcA/UDP-GalNAc dual transporter encoded by solute carrier family 35 member D1 (*SLC35D1*) incorporates both UDP-GlcA and UDP-GalNAc from the cytosol into endoplasmic reticulum (Muraoka et al., 2001). The *Slc35d1*-deficient mouse showed a lethal form of skeletal dysplasia associated with severe shortening of limbs, abnormal facial structures, a decreased proliferating zone with round chondrocytes, scarce matrices, and reduced CS but not HS in long bones (Hiraoka et al., 2007). Furthermore, schneckenbecken dysplasia characterized by perinatally lethal skeletal dysplasia is caused by mutations in *SLC35D1* (Hiraoka et al., 2007). These findings indicate that CS chains and/or CS-PGs are indispensable for early embryonic as well as skeletal development, and that the mutant mouse can be utilized to explore the pathogenic and therapeutic approaches for human schneckenbecken dysplasia.

## Knockout and Mutant Mice of Biosynthetic Enzymes for CS/DS Backbones

### Xylt1 and Xylt2

XYLT1 encoded by *XYLT1* transfers Xyl to specific serine residues in core proteins of PGs from UDP-Xyl as a donor substrate in the Golgi apparatus (Figure 2) (Götting et al., 2000; Schön et al., 2006; Pönighaus et al., 2007). The *Xylt1* mutant *pug*, which is generated by *N*-ethyl-*N*-nitrosourea, and has the substitution Trp932Arg, showed lower XYLT activity in chondrocytes from the mutant than the wild-types, thereby decreasing the production of GAGs in cartilage (Mis et al., 2014). It should be noted that a defect in XYLT1 may affect the biosyntheses of not only CS/DS but also HS, because the linker region tetrasaccharide GlcA-Gal-Gal-Xyl- is common to CS, DS, and HS (Supplemental Figure S1). Moreover, *pug* mutants showed phenotypes including reduced limb, humerus, femur, radius, ulna, tibia, and fibula lengths, and the normal proliferation as well as promotion of premature maturation of chondrocytes, which suggests a general defect in endochondral ossification, resulting in dwarfism. These skeletal abnormalities may be caused by an up-regulation of Indian hedgehog signaling but not FGF signaling (Mis et al., 2014). In fact, mutations in human *XYLT1* cause Desbuquois dysplasia type 2 characterized by severe pre- and postnatal growth retardation, a short stature, joint laxity, and the dislocation of large joints (Bui et al., 2014). Thus, the *pug* mutant mouse is available to help understand the pathogenic mechanism and development of treatment for human Desbuquois dysplasia type 2.

XYLT2 encoded by *XYLT2* also transfers Xyl to specific serine residues in core proteins of PGs from UDP-Xyl as a donor substrate in the Golgi apparatus (Götting et al., 2000; Schön et al., 2006; Pönighaus et al., 2007). The *Xylt2*-deficient mouse exhibited liver abnormalities including biliary tract hyperplasia, liver fibrosis, and biliary cysts, as well as renal abnormalities including dilated tubules, intestinal fibrosis, increase of the renal weight, and hydronephrosis (Condac et al., 2007). Furthermore, it was demonstrated that there is an 86% reduction in HS disaccharides from the liver of *Xylt2*-deficient mice compared with wild-type mice, and a lack of the GAG side chain of decorin, which is a DSPG, in both the liver and kidney of *Xylt2*-deficient mice. The defect in XYLT2 may affect the biosyntheses of not only CS/DS but also HS, because the linker region tetrasaccharide, GlcA-Gal-Gal-Xyl-, is common to CS, DS, and HS (Supplemental Figure S1). However, normal levels of renal CS as well as HS in *Xylt2*-deficient mice were detedcted (Condac et al., 2007). These findings suggest that the residual HS observed in liver from *Xylt2*-deficient mice may be sufficient for hepatocellular differentiation as well as proliferation, but not maturation, and that renal development requires decorin, the DS side chain, or other DSPGs. Homozygous mutations in *XYLT2* cause spondyloocular syndrome that is characterized by retinal detachment, amblyopia, nystagmus, hearing loss, heart septal defects, bone fragility, and mild learning difficulties (Munns et al., 2015). However, patients with predicted null mutations in *XYLT2* did not show polycystic disease. Hence, XYLT1 may compensate for the loss-of-function mutation of XYLT2 in the human liver as well as kidney.

The *Xylt2*-deficient mouse also showed reductions in the size and number of adipocytes, glucose intolerance, and insulin resistance, as well as an increase in serum triglycerides as compared with wild-type mice (Sivasami et al., 2019). Moreover, elevations of interleukin-6 and interleukin-1β, which are proinflammatory M1 cytokines, and the upregulation of TGFβ signaling that inhibits adipogenesis in preadipocyte cells, result in the inflammation of adipose tissues. It was demonstrated that adipose-derived stem cells showed impaired adipogenic differentiation in *Xylt2*-deficient mice, and that maturation of endothelium from gonadal fat tissue was reduced, thereby increasing adipogenic precursors. These findings suggest that the GAG decrease caused by a defect in XYLT2 leads to reduced steady state adipose tissue stores, which is a unique lipodystrophic model.

### Fam20b

Xyl 2-*O*-kinase encoded by *FAM20B* transfers a phosphate group to the Xyl residue in the linkage region from ATP as a donor substrate in the Golgi apparatus (Koike et al., 2009). Conditional knockout (cKO) of *Fam20b* (*Osr2-Cre;Fam20B* ^*flox/flox*^) in the joint cartilage, palate mesenchyme, and metanephric mesenchyme-derived glomeruli tissues, showed that chondrocytes overproliferated but underdifferentiated, and failed to initiate ossification on the popliteal side of the secondary ossification center (Ma et al., 2016). Furthermore, the gain-of-functions of bone morphogenetic protein (BMP) as well as WNT, and the down-regulation of Indian hedgehog, which coordinates chondrocyte proliferation and maturation, were detected in the cartilage of *Fam20b* cKO (Ma et al., 2016). These phenotypes lead to chondrosarcoma in the knee joint and marked defects of postnatal ossification in long bones. However, no significant changes in FGF and TGF-β signaling in *Fam20b* cKO mice were detected. Taken together, the FAM20B-catalyzed PGs are essential for chondrocyte differentiation and maturation, as well as subsequent ossification.

*Wnt1-Cre;Fam20b* ^*flox/flox*^ cKO mice, which were deficient in *Fam20b* in the neural crest and midbrain, died immediately after birth due to complete cleft palates (Liu et al., 2018). Moreover, the *Fam20b* cKO mice showed tongue elevation, micrognathia, microcephaly, suture widening, and reduced mineralization in the calvaria, facial bones, and temporomandibular joint (Liu et al., 2018). These findings suggest that GAG side chains of PGs formed by catalysis of FAM20B are necessary for the morphogenesis and mineralization of the craniofacial complex.

*Col1a1-Cre;Fam20B* ^*flox/flox*^ cKO mice, which were deficient in *Fam20b* in osteoblasts, showed apparent postnatal growth retardation, a shorter tail and spine, and the spinal curvature, resulting in severe kyphosis (Saiyin et al., 2019). Furthermore, *Fam20B* cKO mice showed marked intervertebral disc defects associated with malformation of the peripheral annulus fibrosus, which resulted from the fibrosus tissue transforming to cartilage-like tissue. Not only CS but also HS were reduced in the annulus fibrosus from *Fam20B* cKO mice. TGF-β signaling required for the development and maintenance of the annulus fibrosus and intervertebral disc, was not activated in *Fam20B* cKO mice. MAPK signaling was also modified in cKO mice, *i.e*., increases in phospho-P38 and phospho-ERK but decreases in phospho-JNK (Saiyin et al., 2019). These findings indicate that FAM20B-mediated PGs may play an essential role in annulus fibrosus development through regulating TGF-β and MAPK signaling pathways.

*K14-Cre;Fam20B* ^*flox/flox*^ cKO mice, which were deficient in *Fam20b* in the dental epithelium, showed supernumerary tooth formation. Reductions in CS and HS in the dental epithelium attenuated FGFR2b as well as WNT signalings in the initial stage and later cap stage, respectively, of tooth development (Wu et al., 2020). These findings suggest that FAM20B-catalyzed GAG biosynthesis on PGs regulates the number of murine teeth through FGFR2b signaling in the initial stage of tooth development.

### B3gat3

GlcAT-I encoded by *B3GAT3* transfers the 4th sugar residue in the linker region tetrasaccharide GlcA-Gal-Gal-Xyl from UDP-GlcA to Gal-Gal-Xyl-*O*-serine (Figure 2) (Kitagawa et al., 1998). The *B3gat3*-deficient mice showed embryonic lethality before the 8-cell stage due to the failure of cytokinesis (Izumikawa et al., 2010). Moreover, neither CS nor HS was detected in blastocysts from *B3gat3*-deficient mice (Izumikawa et al., 2010). The defect in B3GAT3 may affect the biosynthesis of not only CS/DS but also HS, because the linker region tetrasaccharide GlcA-Gal-Gal-Xyl- is common to CS, DS, and HS (Supplemental Figure S1). Interestingly, treatment of 2-cell embryos with chondroitinase, which is a bacterial eliminase acting specifically on CS, resulted in embryonic lethality between 2- and 8-cell stages, but treatment with heparitinase, a bacterial eliminase acting specifically on HS, showed no lethality (Izumikawa et al., 2010). *Ext1*- or *Ext2*-deficient mice that lack HS developed normally until embryonic day 6.5 (Lin et al., 2000; Stickens et al., 2005). EXT1 and EXT2 have both HS-GlcAT-II and α-1,4*N*-acetylglucosaminyltransferase-II activities, which are required for biosynthesis of HS chains (Lind et al., 1998; McCormick et al., 1998) (Supplemental Figure S1). *Caenorhabditis elegans* synthesizes chondroitin, non-sulfated CS, which is required for normal cell division and cytokinesis in an early developmental stage (Mizuguchi et al., 2003; Izumikawa et al., 2004). These findings suggest that abnormal cytokinesis in *B3gat3*-deficient mice may be attributed to deficiency in CS, but not HS.

Embryonic stem cells derived from *B3gat3*-deficient mice completely lost both CS and HS, and failed to differentiate into multiple lineages (Izumikawa et al., 2014). Degradation of CS on wild-type embryonic stem cells by treatment with chondroitinase had effects on the formation of embryonic bodies, which is *in vitro* differentiation by free-floating aggregates of the embryonic stem cells, whereas treatment with heparitinase showed no effects on the development of embryonic bodies. Furthermore, the exogeneous addition of CS-A or CS-E polysaccharides to embryonic bodies derived from *B3gat3*-deficient mice rescued the differentiation of these cells into primitive endodermal cells in a culture assay (Izumikawa et al., 2014). The interaction of CS with E-cadherin regulates the Rho signaling pathway, which leads to the control of differentiation of embryonic stem cells (Izumikawa et al., 2014). These findings suggest that CS contributes to the integrity of embryonic stem cells via interaction with E-cadherin.

### Csgalnact1 and Csgalnact2

*N*-Acetylgalactosaminyltransferase (GalNAcT) encoded by *CSGALNACT1* or *CSGALNACT2* transfers a GalNAc residue from UDP-GalNAc to GlcA-Gal-Gal-Xyl-*O*-serine and [GlcA-GalNAc]_n_ (Figure 2) (Uyama et al., 2002; 2003). *Csgalnact1*-deficient mice showed a short body length and small body weight, caused by shortening of the limbs and axial skeleton, and a thinner growth plate in cartilage than wild-type mice (Watanabe et al., 2010; Sato et al., 2011). The level of CS disaccharides in the cartilage from the *Csgalnact1*-deficient mice was reduced to half of that in the wild-type (Watanabe et al., 2010; Sato et al., 2011). These findings indicate that CSGALNACT1 and/or CS-PG is necessary for the differentiation and maturation of cartilage.

*Csgalnact1*-deficient mice also showed impaired intramembranous ossification, resulting in a shorter face, and higher and broader calvaria (Ida-Yonemochi et al., 2018). Protein levels of Wnt3a and β-catenin were decreased in the mesenchymal tissues of calvaria, and collagen fibers were irregular, thick, and aggregated in the calvaria and scalp from *Csgalnact1*-deficient mice, which causes skull abnormalities (Ida-Yonemochi et al., 2018). Furthermore, *Csgalnact1*-deficient mice were characterized by malocclusion, abnormal eyes, skin hyperextension, severe scoliosis, joint laxity, and reduction of CS in skin, muscle, tendon, and bone, which are similar to the hallmarks of Ehlers-Danlos syndrome in humans. Loss of CSGALNACT1 may cause disturbance of DS biosynthesis, because chondroitin is a precursor for DS. Musculocontractural Ehlers-Danlos syndrome is caused by a defect in DS (Malfait et al., 2017; 2020).

*Csgalnact1*-deficient mice showed better recovery after spinal cord injury than wild-type mice, based on a footfall test, footprint test, and electromyography, because of the promotion of axonal regeneration (Takeuchi et al., 2013). On the other hand, *Csgalnact2*-deficient mice have not been demonstrated to show such promotional activity. After spinal cord injury, the biosynthesis of CS is promoted and resultant CS inhibits axonal regeneration as a barrier-forming molecule (Carulli et al., 2005). However, the promotion of CS biosynthesis is lower in *Csgalnact1*-deficient mice than in wild-type mice (Takeuchi et al., 2013). Interestingly, an increase of HS was detected in association with up-regulations of *Ext1*, *Ext2*, and *Extl3* mRNAs that encode glycosyltransferases responsible for HS biosynthesis (Takeuchi et al., 2013). HS has been reported to promote axonal growth and regulate axon guidance (Yamaguchi, 2001). Thus, the decrease and increase of CS and HS, respectively, in *Csgalnact1*-deficient mice resulted in better recovery from spinal cord injury than in wild-type mice.

CS-PG is a major component in perineuronal nets, which are unique extracellular matrix structures that wrap around neurons during development and control plasticity in the central nervous system (Sorg et al., 2016). *Csgalnact1*-deficient mice showed a significant decrease in CS in the cerebrum, diencephalon, spinal cord, and visual cortex (Yoshioka et al., 2017). Furthermore, *Csgalnact1*-deficient mice showed a significantly greater total distance traveled than wild-type mice in the open field test, which measures voluntary activity in a novel environment. *Csgalnact1*-deficient mice manifested much larger responses than wild-type mice in an acoustic startle test, which can measure reflex movement in response to a sudden loud sound stimulus (Yoshioka et al., 2017). These findings suggest that CS generated by CSGALNACT1 may affect the formation of perineuronal nets as well as behaviors of mice.

*Csgalnact1*-deficient mice were characterized by a reduction in CS in the visual cortical area and impaired ocular plasticity, which is caused by a decrease of Otx2 accumulation (Hou et al., 2017). CS binds to Otx2 in perineuronal nets, and promotes uptake of Otx2 into parvalbumin-expressing basket cells, thereby terminating the critical period for plasticity (Miyata and Kitagawa, 2015). These findings indicate that CS and CS-PGs are required for the critical period for plasticity in the visual cortex.

*Csgalnact1*-deficient mice with experimentally induced autoimmune encephalomyelitis showed milder symptoms including lower cell infiltration, proliferation, and productions of interleukin-6 and interferon-γ than those in the wild-type (Inada et al., 2021). These findings suggest that CS side chains of PGs may be associated with autoimmune encephalomyelitis and potential therapeutic targets for neuroimmunological diseases.

*Csgalnact2*-deficient mice exhibited normal development, fertility, growth rates, and skeletal formation (Shimbo et al., 2017). These findings suggest that loss of functions of CSGALNACT2 might be compensated for by CSGALNACT1.

Mice with double KO of *Csgalnact1* and *Csgalnact2* died during the postnatal stage due to respiratory failure (Shimbo et al., 2017). Furthermore, the double KO mice exhibited severer phenotypes including short humeral and tibial lengths compared with *Csgalnact1-*or *Csgalnact2*-deficient mice. The total CS disaccharides in rib cartilage from *Csgalnact1*-KO, *Csgalnact2*-KO, and double KO mice were reduced to ∼74, ∼99, and ∼40%, compared with that of the wild-type, respectively (Shimbo et al., 2017).

Approximately 80% of *Col2a1-Cre; Csgalnact1* ^*flox/—*^ *; Csgalnact2* ^*flox/—*^ double cKO mice, which were deficient in both *Csgalnact1* and *Csgalnact2* in chondrocytes, immediately died after birth because of respiratory failure, and the remaining ∼20% of the double KO mice could start spontaneous respiration (Shimbo et al., 2017). They were characterized by a lower body weight, severer dwarfism, and lower proliferation of chondrocytes than control mice.

These data indicate that CS synthesized by CSGALNACT1 and CSGALNACT2, may be required for pulmonary and skeletal development during embryogenesis.

### Chsy1

GalNAcT-II and glucuronyltransferase-II (GlcAT-II) encoded by *CHSY1* transfer GalNAc and GlcA residues from UDP-GalNAc and UDP-GlcA to [GlcA-GalNAc]_n_ or [GalNAc-GlcA]_n_, respectively (Figure 2) (Kitagawa et al., 2001b). *Chsy1*-deficient mice were characterized by chondrodysplasia, progression of the bifurcation of digits, delayed endochondral ossification, and reduced bone density (Wilson et al., 2012). Furthermore, a decrease in 4-*O*-sulfation and increases in 6-*O*-sulfation as well as non-sulfated GalNAc residues were detected in the cartilage of *Chsy1*-deficient mice. The up-regulation of transcriptional target of Hedgehog, *Gli1*, was detected in embryonic fibroblast cultures from *Chsy1*-deficient mice (Wilson et al., 2012). Moreover, a brachymorphic mouse with mutation in *Papss2* also showed low sulfated CS in the cartilage, and its Hedgehog signaling was attenuated (Orkin et al., 1976; Cortes et al., 2009). These findings indicate that CS and Hedgehog protein may coordinately modulate bone development.

Small with kinky tail (*skt*) mutant mice spontaneously arose at the Jackcon Laboratory with recessive mutation (Lane, 1988). The *skt* mutant was caused by a 27-kb deletion containing *Chsy1* (Macke et al., 2020). The *skt* mutant mice showed reduced CS in the retina as well as hippocampus compared with heterozygous deficient mice, an increase in a number of empty spaces surrounding cells in the cornu ammonis 1, 2, and 3 hippocampal subfields compared with control mice, decreased neutrophils in bone marrow as well as macrophages in both the bone marrow and spleen, and age-dependent retinal changes including progressive photoreceptor cell degeneration with an increase of glial fibrillary acidic protein, considered to be a sign of retinal stress (Macke et al., 2020). In contrast, frequencies of monocytic cells and lymphocytic cells such as T-cells, B-cells, and natural killer cells, did not appear to be consistently altered in the *skt* mutant mice compared with heterozygous controls. These findings suggest that CS constructed by CHSY1 regulates the development of the hippocampus, retina, neutrophils, and macrophages.

### Chsy3

CHSY3 also has a dual enzymatic activity with β1,3-GlcA transferase and β1,4-GalNAc transferase on its amino- and carboxy-terminal sides, respectively (Yada et al., 2003a; Izumikawa et al., 2007). *Chsy3*-deficient mice showed a shorter body length than the wild-type after 4 weeks old, a reduction of CS in disc tissues, and intervertebral disc degeneration such as a narrowed disc height, loss of the nucleus pulposus, and unclear demarcation between the nucleus pulposus and annulus fibrosus (Wei et al., 2020). Furthermore, the Hippo signaling pathway, which is regulated by a kinase of the Sterile-20 family and activates the suppressor Warts (Zheng and Pan, 2019), was significantly downregulated. The activation of Yap1, which is a transcriptional coactivator as well as a negative regulator of the Hippo pathway, and is involved in intervertebral disc degeneration (Chen et al., 2019), was mainly affected in nucleus pulposus cells from *Chsy3*-deficient mice (Wei et al., 2020). These findings suggest that CS activates Yap signaling and spontaneous intervertebral disc degeneration.

### Chpf

Chondroitin polymerizing factor encoded by *CHPF* exhibits an enzymatic activity to polymerize the disaccharide region of CS in concert with CHSY1 *in vitro* (Kitagawa et al., 2003). Since CHPF has a dual enzymatic activity of β1,3-GlcA transferase and β1,4-GalNAc transferase, it was also designated as CHSY2 (Yada et al., 2003b). Although *Chpf*-deficient mice showed no obvious abnormalities, the femur and tibia lengths were slightly reduced, and the chain length of CS was shorter in cartilage than in wild-type mice (Ogawa et al., 2012). These findings indicate that other CHSY family proteins, CHPF2, CHSY1, and/or CHSY3, might compensate for the activity of CHPF.

### Chpf2

CHPF2 also has a dual enzymatic activity of β1,3-GlcA transferase and β1,4-GalNAc transferase, and has been designated as CHSY3 or CSGlcA-T (Gotoh et al., 2002; Izumikawa et al., 2008). *Chpf2*-deficient mice have been registered in the knockout mouse library, and their anomalies in the bone and heart were reported without detailed analyses (Tang et al., 2010). Further investigation is required for elucidation of the *in vivo* function of CHPF2.

### Chst3

C6ST1 encoded by *carbohydrate sulfotransferase 3* (*CHST3*) transfers a sulfate group from PAPS to the C-6 hydroxy group of GalNAc residues in the CS repeating disaccharide region, [GlcA-GalNAc]_n_ (Figure 3) (Fukuta et al., 1995, 1998; Uchimura et al., 1998). *Chst3*-deficient mice showed a loss of 6-*O*-sulfated disaccharide units such as the C-unit, GlcA–GalNAc(6-*O*-sulfate), and D-unit, GlcA(2-*O*-sulfate)–GalNAc(6-*O*-sulfate), in the spleen, cartilage, and brain (Uchimura et al., 2002), although brain development seems to be normal in *Chst3*-deficient mice. *Chst3* was not expressed in the thymus (Uchimura et al., 1998), where naive T-cells differentiate, and the proportion of CD4^+^/CD8^–^ and CD4^–^/CD8^+^ cells in the thymus from *Chst3*-deficient mice did not change (Uchimura et al., 2002). However, the number of naive T-lymphocytes decreased (Uchimura et al., 2002). These findings indicate that survival, retention, and/or emigration of naive T lymphocytes was affected in the spleen of the *Chst3*-deficient mice, rather than that of thymocytes.

After axotomy of nigrostriatal axons, *Chst3*-deficient mice exhibited fewer regenerating axons and more axonal retraction than wild-type mice (Lin et al., 2011), although repair of the median and ulnar nerves was similar between wild-type and *Chst3*-deficient mice after peripheral nerve injury. Increases in the expression of *Chst3* and proportion of the 6-*O*-sulfated structure have been demonstrated in glial scars after cortical injury (Properzi et al., 2005). These findings suggest that the suppression of 6-*O*-sulfation in CS after injury of the central nervous system prevents axons to regenerate.

*Chst3*-transgenic mice with an increase in 6-*O*-sulfation of the brain CS showed loss of perineuronal nets in the brain, leading to the continuance of the critical period for cortical plasticity (Miyata et al., 2012). Furthermore, Otx2, which is a homeoprotein and regulates ocular dominance plasticity via its effects on maturation of parvalbumin-expressing interneurons (Sugiyama et al., 2008), diffused and reduced at the surrounding parvalbumin-expressing interneurons in *Chst3*-transgenic mice (Miyata et al., 2012). These findings indicate that 6-*O*-sulfation of CS at perineuronal nets in the brain regulates the critical period for cortical plasticity by maturation of parvalbumin-expressing interneurons.

*Chst3*-deficient mice presented with a hyperthickened epidermis, enhanced proliferation, and altered differentiation of basal keratinocytes, thereby impairing the epidermal permeability barrier function (Kitazawa et al., 2021). Furthermore, the 6-*O*-sulfated CS directly binds to epidermal growth factor receptor (EGFR), leading to the blockade of EGFR signaling (Kitazawa et al., 2021). The *Chst3*-deficient mice had a thicker epidermis and increased levels of acute inflammation including erythema, scaling, and skin induration, compared with wild-type mice when psoriasis was induced by imiquimod (Kitazawa et al., 2021). These findings indicate that the 6-*O*-sulfated CS repress proliferation of keratinocytes and progression of psoriasis in the skin.

### Chst11

C4ST1 encoded by *carbohydrate sulfotransferase 11* (*CHST11*) transfers a sulfate group from PAPS to the C-4 hydroxy group of GalNAc residues in the CS repeating disaccharide region, [GlcA-GalNAc]_n_ (Figure 3) (Hiraoka et al., 2000; Yamauchi et al., 2000). *Chst11*-deficient mice showed a more than 90% reduction of the 4-*O*-sulfated disaccharide unit in the growth plate compared with the wild-type (Klüppel et al., 2005). Furthermore, they exhibited severe dwarfism, multiple skeletal abnormalities including a small rib cage, a kinked vertebral column, severely shortened limbs, a dome-shaped skull, reduction in Alcian blue staining in cartilage, and fatality within 6 h of birth with severe respiratory distress (Klüppel et al., 2005). In the *Chst11*-deficient embryos, chondrocyte differentiation was affected during morphogenesis of the cartilage growth plate because of upregulation of TGFβ signaling with concomitant downregulation of BMP signaling, but not Indian hedgehog signaling (Klüppel et al., 2005), although mesenchymal aggregation and cartilage primordium formation were normal. These findings suggest that CS 4-*O*-sulfation and C4ST1 are required for embryonic development and morphogenesis of the cartilage growth plate by modulation of signaling pathways.

### Chst15

GalNAc4S-6ST encoded by *carbohydrate sulfotransferase 15* (*CHST15*) transfers a sulfate group from PAPS to the C-6 hydroxy group of GalNAc4-*O*-sulfate residues in the CS repeating disaccharide region, [GlcA-GalNAc(4-*O*-sulfate)]_n_ (Figure 3) (Ohtake et al., 2001). *Chst15*-deficient mice showed complete loss of GalNAc 4- and 6-*O*-disulfated structure (E-unit) in CS/DS from the tissues examined, including the cerebrum, cerebellum, heart, lung, liver, spleen, kidney, thymus, stomach, small intestine, large intestine, mesentery, testis, whole embryo, and bone marrow-derived mast cells, suggesting that GalNAc4S-6ST encoded by *Chst15* is the sole enzyme responsible for the biosynthesis of GalNAc 4- and 6-*O*-disulfated structure (Ohtake-Niimi et al., 2010). Furthermore, *Chst15*-deficient mice were fertile, showed normal development, exhibited weak staining of bone marrow-derived mast cells with May Grünwald-Giemsa, showed an increase of empty granules in bone marrow-derived mast cells, and presented lower activities of carboxypeptidase A as well as tryptase from bone marrow-derived mast cells (Ohtake-Niimi et al., 2010). These findings suggest that GalNAc 4- and 6-*O*-disulfated structure in CS/DS-PGs may be involved in the storage of these proteases in the granules of mast cells.

*Chst15*-deficient mice also exhibited impairment of osteoblast differentiation leading to be low bone mass (Koike et al., 2015). Liver fibrosis induced by CCl_4_ was enhanced in these mice (Habuchi et al., 2016). These findings indicate that GalNAc4S-6ST and/or E-disaccharide unit-containing CS, [GlcA-GalNAc(4-, 6-*O*-disulfates)], may be a therapeutic target for osteopenia, osteoporosis, and fibrosis. However, GalNAc 4- and 6-*O*-disulfated structure was not necessary for binding with semaphoring 3A in the perineuronal nets of brain (Nadanaka et al., 2020).

### Dse and Dsel

DS-epimerase encoded by *DSE* or *DSEL* converts GlcA into IdoA by C5-epimerization of GlcA residues in the CS repeating disaccharide region, [GlcA-GalNAc]_n_ (Figure 2) (Maccarana et al., 2006; Pacheco et al., 2009). *Dse*-deficient mice exhibited a smaller body weight, reductions in IdoA-containing structures in the skin, thicker collagen fibrils in the dermis and hypodermis, kinked tails, impairment of directional migration of aortic smooth muscle cells, and defects in the fetal abdominal wall, exencephaly, and spina bifida (Maccarana et al., 2009; Bartolini et al., 2013; Gustafsson et al., 2014). Dse and/or DS may be indispensable for normal development and formation of collagen fibrils.

*Dsel*-deficient mice had no anatomical, histological, or morphological abnormalities (Bartolini et al., 2012). Furthermore, *Dsel*-deficient mice exhibited reduced epimerase activity in the skin (24% reduction), lung (34%), liver (38%), spleen (44%), kidney (55%), and brain (89%) compared with those in the wild-type mouse tissues (Bartolini et al., 2012). Consistent with this result, IdoA contents of CS/DS chains from the neonatal brain and kidney were reduced to 87 and 62% of wild-type mice, respectively (Bartolini et al., 2012). Brain from *Dsel*-deficient mice showed normal extracellular matrix features by immunohistological staining. DSE may compensate for the function of DSEL.

Double knockout mice of *Dse* and *Dsel* exhibited perinatal lethality with an umbilical hernia, exencephaly, a kinked tail, and complete loss of DS, suggesting that DS plays an important role in embryonic development as well as perinatal survival (Stachtea et al., 2015).

### Chst14

D4ST1 encoded by *carbohydrate sulfotransferase 14* (*CHST14*) transfers a sulfate group from PAPS to the C-4 hydroxy group of GalNAc residues in the repeating disaccharide region of DS, [IdoA-GalNAc]_n_ (Figure 3) (Evers et al., 2001; Mikami et al., 2003). *Chst14*-deficient mice showed a smaller body mass, reduced fertility, kinked tail, and increased skin fragility compared with wild-type littermates (Akyüz et al., 2013). Moreover, in *Chst14*-deficient mouse skin, the amount of DS was markedly decreased with elevation of the level of CS, which is a precursor chain of DS. These phenotypes of *Chst14*-deficient mice were considerably similar to those of *Dse*-deficient mice (Maccarana et al., 2006). In addition to both enzymes involving the biosynthesis of DS, it has been reported that 4-*O*-sulfated GalNAc residues in DS chains prevent back-epimerization by DSE *in vitro* (Malmström, 1984). Furthermore, DSE and CHST14 forms heterocomplex, but not DSEL, which is necessary to build longer IdoA-containing chains (Tykesson et al., 2018). Therefore, the cooperation of both enzymes by heterocomplex is required for the formation of repeating disaccharide, [GalNAc(4S)–IdoA], in DS.

Its skin tensile strength was significantly decreased compared with that in wild-type mice, and the collagen fibrils were oriented in various directions to form disorganized collagen fibers in the reticular layer (Hirose et al., 2021). Rod-shaped linear GAG chains were found to be attached at one end to collagen fibrils and protruded outside of the fibrils in the *Chst14*-deficient mice, in contrast to those being round and wrapping the collagen fibrils in wild-type mice (Hirose et al., 2021). These findings suggest that the DS side chain of decorin is necessary for assembly of decorin-PG with collagen, and maintenance of the skin strength.

CRISPR/Cas9-genome engineered *Chst14*-deficient mice exhibited common growth impairment and skin fragility similar to the conventional knockout mice of *Chst14* (Nitahara-Kasahara et al., 2021a). In addition, CRISPR/Cas9-genome engineered *Chst14*-deficient mice showed decreased DS in muscle, thoracic kyphosis, and myopathy-related phenotypes including variation in fiber size and spread of the muscle interstitium, as well as diffuse localization of decorin in the spread endomysium of skeletal muscle, which caused the lower grip strength and decreased exercise capacity, compared with wild-type and heterozygous mutant mice (Nitahara-Kasahara et al., 2021a; 2021b). The CRISPR/Cas9-engineered *Chst14*-mutant mouse is a useful model for musculocontractural Ehlers-Danlos syndrome caused by mutations in CHST14 (Dündar et al., 2009; Malfait et al., 2010; Miyake et al., 2010; Voermans et al., 2012; Kosho et al., 2019; Malfait et al., 2020).

*Chst14*-deficient mice are sometimes perinatally lethal (Yoshizawa et al., 2018). Their placenta showed immaturity such as a reduced weight of the placenta, alteration in the vascular structure with ischemic and/or necrotic-like change, an abnormal structure of the basement membrane of capillaries in the placental villus, and significantly decreased DS (Yoshizawa et al., 2018). These findings suggest that DS may be essential for placental vascular development.

Cultured Schwann cells from dorsal roots and nerves, cerebellar neurons, and motoneurons of *Chst14*-deficient mice exhibited longer cell processes compared with those from wild-type cells (Akyüz et al., 2013). Schwann cells from *Chst14*-deficient mice had a higher proliferation rate. Moreover, the values for the foot-base and heel-tail angles in *Chst14*-deficient mice showed better recovery than those in wild-type mice at each time-point between 1 and 12 weeks after femoral nerve injury (Akyüz et al., 2013). These findings indicate that *Chst14* partially controls inhibitory functions during neural development and recovery from nerve injury.

Neurospheres from *Chst14*-deficient, but not *Chst11*-deficient mice exhibited fewer numbers and larger diameters than those from wild-types (Bian et al., 2011). This was caused by impairments of self-renewal and proliferation, but neither apotosis nor migration, of neural stem cells *in vitro* as well as *in vivo* (Bian et al., 2011). The expression level of GLAST but not Nestin, which are markers of radial glial cells and neurons, respectively, was increased in neurospheres from *Chst14*-deficient mice. These findings suggest that DS-PGs play important roles in the proliferation and differentiation of neural stem cells.

## Knockout and Mutant Mice of Catabolism of the Reaction Products of Donor Substrates, UDP and PAP

### Cant1

Most glycosyltransferases utilize UDP-sugar as a donor substrate, which is converted to UDP after the reaction in the endoplasmic reticulum or Golgi apparatus. The UDP is hydrolyzed to UMP by 5′-diphosphatase encoded by *CANT1* (Failer et al., 2002; Smith et al., 2002). *Cant1*-deficient mice exhibited moderate kyphosis, a decrease in both the length and width of tibiae, femurs, and ilium, delta phalanx, a defect in endochondral ossification, and reduction in GAGs in chondrocytes (Paganini et al., 2019). Furthermore, the phenotypes of the *Cant1*-knockout mouse were similar to those of a *Cant1* knock-in mouse with an Arg302His substitution in the catalytic domain (Huber et al., 2009), which corresponds to the human mutation in patients with Desbuquois dysplasia characterized by a short stature, round face, progressive scoliosis, and joint laxity (Paganini et al., 2019). *Cant1*-deficient mice generated by the CRISPR/Cas9 system also exhibited a lower body weight, short stature, thoracic kyphosis, delta phalanx, reduction in GAG content in growth plate cartilage, and impairment of differentiation of chondrocytes (Kodama et al., 2020).

These findings suggest that CANT1 and/or hydrolysis of UDP to UMP may be necessary for the metabolism of GAGs and that it affects the maturation of chondrocytes in the cartilage growth plate. Accumulation of UDP may inhibit the activity of glycosyltransferases involved in the biosynthesis of GAGs. The lack of UMP may inhibit the incorporation of UDP-sugars from the cytosol into the endoplasmic reticulum and Golgi apparatus through antiporters, nucleotide sugar transporters. Further biochemical analyses of the cellular pathways will be crucial in order to elucidate the molecular basis of CANT1 deficiency as well as Desbuquois dysplasia.

### Bpnt2

Most sulfotransferases utilize PAPS as a donor substrate, which is converted to PAP after the reaction in the cytosol as well as Golgi apparatus. PAP is hydrolyzed to 5′-AMP by PAP 3′-phosphatase encoded by *BPNT1* and *BPNT2/IMPAD1* in the cytosol and Golgi apparatus, respectively (Frederick et al., 2008; Hudson et al., 2013). The gene trap *Bpnt2*-deficient mice are neonatally or embryonically lethal, and showed reduction of the limb length, shortening of the snout and lower limbs, reduced sternal length, and diminished rib spacing (Frederick et al., 2008). Furthermore, a marked decrease in chondroitin 4-*O*-sulfate and an increase in non-sulfated chondroitin were detected in the cartilage, lung, and embryos of *Bpnt2*-deficient mice. Although significant changes in the amount and sulfation modification of HS were not observed in the embryos from mutant mice, the degree of sulfation of HS was slightly decreased in the lung (Frederick et al., 2008). These findings indicate that BPNT2 and/or hydrolysis of PAP to 5′-AMP may be necessary for the metabolism of sulfation of GAGs and that it affects skeletal development. The accumulation of PAP may inhibit sulfotransferases involved in the biosynthesis of GAGs. The lack of 5′-AMP may inhibit the incorporation of PAPS from the cytosol into Golgi apparatus through an unidentified antiporter(s).

## Knockout Mice of Golgins

### Gorab

Golgins comprise a family of vesicle-tethering proteins at the Golgi apparatus (Witkos and Lowe, 2017; Lowe, 2019). The vesicle-bound cargo tethers to the Golgi apparatus, which triggers membrane fusion. Various golgins are localized to distinct regions of the Golgi apparatus, and their ability to tether transported vesicles selectively is necessary for the specificity of vesicle traffic in the secretory pathway. Because the biosynthesis of GAG side chains on PGs is achieved in the endoplasmic reticulum and Golgi apparatus, some golgins are most likely involved in the transport of PGs.

*GORAB* encodes a Rab6-interacting Golgi protein, and its mutations cause human genetic disorder, gerodermia osteodysplastica, which is characterized by skin laxity and early-onset osteoporosis (Hennies et al., 2008). Mutant mice of *Gorab* have been generated, with fully and conditionally inactivated mesenchymal progenitor cells (*Prx1*-cre), pre-osteoblasts (*Runx2*-cre), and late osteoblasts/osteocytes (*Dmp1*-cre), respectively (Chan et al., 2018). The *Gorab* full-knockout mice (*Gorab* ^Null^) were neonatal lethal, and showed disorganized collagen fibrils (Chan et al., 2018). The *Gorab* conditional-knockout mice, *Gorab* ^Prx1^ and *Gorab* ^Runx2^, exhibited thinned, porous cortical bone and spontaneous fractures (Chan et al., 2018), which were also observed in a patient with gerodermia osteodysplastica (Hennies et al., 2008). Furthermore, the level of DS, but not CS or HS, was decreased in skin and cartilage from *Gorab* ^Null^ mutants. The glycanation of DS-proteoglycans, biglycan and decorin, in skin and bone may be reduced (Chan et al., 2018). The Golgi apparatus compartment of cultured fibroblasts from *Gorab* ^Null^ mutants showed the accumulation of decorin core protein, but a reduced level of DS, indicating that the newly synthesized decorin core protein accumulates within the Golgi apparatus due to the impairment of DS biosynthesis. However, it remains unclear whether there are anomalies in the transport of decorin core protein or DS-biosynthetic enzymes including DSE as well as D4ST1 to the Golgi apparatus. Taken together, these findings suggest that mutation and/or deficiency of *Gorab* primarily perturbs pre-osteoblasts, and that gerodermia osteodysplastica might be affected by biosynthesis of the DS side chain in proteoglycans and/or transport of decorin core protein in the Golgi compartment.

## Conclusions and Perspectives

Mice deficient in glycosyltransferases or sulfotransferases involved in the biosynthesis of CS/DS demonstrated abnormalities of bone, skin, and nervous systems. These knockout mice with deficiency of *Chst11*, *Chst3*, and *Chst15* have revealed that A, C, and E units in CS chains play essential roles in chondrocyte differentiation, T-cell differentiation, and storage of proteases in mast cells, respectively. Furthermore, *Chst14*-knockout mice revealed that DS-containing iA unit, but not CS-containing A unit, bundles collagen fibrils in skin, which might be dependent on the structural and conformational alteration of CS and DS chains (Casu et al., 1988; Hirose et al., 2021). These findings indicate that specific sulfation modifications as well as conformation of uronic acid in CS/DS are essential for connective tissue and neuronal development.

Recent advances in studies on human genetic disorders in connective tissues have also clarified the biological significance of CS/DS side chains of PGs (Mizumoto et al., 2013, 2017; Mizumoto, 2018; Kosho et al., 2019; Malfait et al., 2020). The clinical halmarks in human diseases caused by deficiency in the biosynthetic enzymes of CS/DS are not always consistent with the phenotypes of knockout mice with deficiency of the corresponding enzymes. This contradiction may be due to residual enzymatic activity in human patients. However, the phenotypes of some null-mutant mice are consistent with human clinical symptoms in patients with mutations in the corresponding gene. Further studies on molecular pathogeneses involving CS and DS chains of PGs are necessary to develop therapeutics and new drugs against these diseases (Table 4).

The biosynthesis of CS/DS-PGs is up-regulated in both tumor stroma and neoplastic cells, resulting in the abundant accumulation of these components in the tumor stroma adjacent to neoplastic cells (Fukatsu et al., 1988; Iozzo et al., 1989; ten Dam et al., 2009; Thelin et al., 2012). Consistent with these observations, up-regulations of gene expressions including glycosyltransferases, epimerases, and sulfotransferases responsible for the biosynthesis of CS/DS (Huang et al., 2021). These findings indicate that CS/DS-PGs contribute to the functions and phenotypes of tumor cells as effectors or modulator macromolecules (ten Dam et al., 2007; Bi et al., 2008; Li et al., 2008; Sugahara et al., 2008; Mizumoto et al., 2012). However, there is little or no report regarding tumor biology of CS/DS using the knockout mice. Further studies on the molecular mechanisms underlying pathological conditions involving CS/DS-PGs using the knockout mice will provide insights into new therapeutic approaches for tumor development (Table 4).
