# Supplementary material for: An Overview of in vivo Functions of Chondroitin Sulfate and Dermatan Sulfate Revealed by Their Deficient Mice
Source: Front Cell Dev Biol. 2021 Nov 24;9:764781. doi: 10.3389/fcell.2021.764781 (PMC8652114; doi:10.3389/fcell.2021.764781)
Supplement: Supplementary file 1 [file DataSheet1.DOCX]

Supplementary Material

# Supplementary Figure

**Supplementary Figure S1. Schematic presentation of the biosyntheses of heparan sulfate and heparin backbones.**

After specific core proteins have been translated, biosynthesis of the common glycosaminoglycan (GAG)-protein linkage region, GlcAβ1-3Galβ1-3Galβ1-4Xylβ1-, where GlcA, Gal, and Xyl stand for D-glucuronic acid, D-galacrose, and D-xylose, respectively, is evoked by XylT, which transfers a Xyl residue from uridine diphosphate (UDP)-Xyl to the specific serine residue(s) at GAG attachment sites. The linker region tetrasaccharide is subsequently constructed by β4-galactosyltransferase-I (GalT-I), GalT-II, and β3-glucuronyltransferase-I (GlcAT-I). The first α1-4-linked *N*-acetyl-D-glucosamine (GlcNAc) residue is then transferred to the GlcA residue in the linker region by α4-*N*-acetylgalactosaminyltransferase-I (GlcNAcT-I), which initiates assembly of the heparan backbone, y resulting in the formation of the repeating disaccharide region, [-4GlcAβ1-4GlcNAcα1-]_n_, by HS-polymerase. Each enzyme and its coding gene are described.

Modification pathways of HS. After formation of the HS backbone, each sugar residue is modified by *N*-deacetylation, *O*-sulfation, and epimerization catalyzed by *N*-deacetylase, sulfotransferases, and C5-epimerase, respectively, as indicated in the figure. The first modifications, *N*-deacetylation and *N*-sulfation, are essential for all subsequent reactions. Next, some GlcA residues adjacent to N-sulfated glucosamine (GlcNS) residues are converted to IdoA residues by GlcA C5-epimerase. Thereafter, sulfation at C-2 of IdoA residues, as well as at C-6 and C-3 of GlcNS and/or GlcNAc residues takes place through the actions of specific sulfotransferases. 2S, 3S, 6S, and NS stand for 2-*O*-sulfate, 3-*O*-sulfate, 6-*O*-sulfate, and 2-*N*-sulfate, respectively.
